# Identification of a Lactylation‐Related Gene Signature and Candidate Biomarkers for Dilated Cardiomyopathy

**DOI:** 10.1111/jcmm.71379

**Published:** 2026-09-25

**Authors:** Xiaoyan Zhi, Miao Zhang, Luyao Xu, Yajing Zhang, Zhijun Zhang, Xiaohui Wang, Li Wang

**Affiliations:** ^1^ Key Laboratory of Cellular Physiology (Shanxi Medical University) Ministry of Education Taiyuan Shanxi Province China; ^2^ Department of Cardiology, Shanxi Bethune Hospital Third Hospital of Shanxi Medical University Taiyuan Shanxi Province China

**Keywords:** candidate biomarkers, dilated cardiomyopathy, *EXT1*, *IER3*, lactylation‐related genes

## Abstract

Dilated cardiomyopathy (DCM) is a leading cause of heart failure. Due to its complex pathogenesis, effective treatment strategies remain limited. Therefore, it is particularly important to identify novel candidate biomarkers from the pathogenesis level. In recent years, lactylation modification plays an important role in various cardiovascular diseases; however, its specific involvement in DCM remains unclear. Therefore, it is of great significance to identify lactylation‐related biomarkers associated with DCM to provide insights for future mechanistic investigations. The DCM gene expression datasets were obtained from the Gene Expression Omnibus (GEO) database. Differentially expressed genes and co‐expression modules were identified using differential expression analysis and weighted gene co‐expression network analysis (WGCNA). Lactylation‐related gene sets (LRGs) were obtained from the MSigDB database. The intersection of these results was further analysed using machine learning algorithms: Least Absolute Shrinkage and Selection Operator (LASSO), Random Forest (RF), and Support Vector Machine (SVM). CIBERSORT algorithm was used to analyse the immune infiltration characteristics of DCM, and DCM was divided into two subtypes by unsupervised consensus clustering. Mendelian randomization (MR) analysis was employed to evaluate potential causal associations between the candidate genes and DCM or heart failure progression. Finally, the candidate genes were verified by the GSE57338 database and β_1_‐AA‐induced heart failure model with DCM characteristics. Through comprehensive transcriptomic analysis integrating WGCNA and LRGs, 38 characteristic genes associated with DCM and lactylation were finally identified. Subsequently, 18 key genes were selected by machine learning. The risk prediction model based on these genes demonstrated good predictive performance for DCM. Immune infiltration analysis revealed dysregulated inflammatory responses in DCM patients, and 18 key genes were significantly related to CD8^+^ T cells, CD4^+^ T cells, neutrophils, and Natural killer T cell infiltration. Unsupervised consensus clustering divided DCM patients into two distinct subtypes (C1 and C2), with marked differences in gene expression. Subtype C1 was characterized by activation of CD8^+^ T cells and Natural Killer T cells, whereas C2 mainly exhibited CD4^+^ T cells and neutrophils activation. Furthermore, Mendelian randomization analysis suggested that *EXT1* may be associated with heart failure, while *IER3* was potentially associated with DCM. Finally, the external validation set GSE57338 and the heart failure model with DCM characteristics were used to verify the two genes identified. The results showed that *EXT1* was up‐regulated and *IER3* was down‐regulated. Based on the deep excavation of public database, combined with machine learning algorithm and Mendelian randomization, this study identified *EXT1* and *IER3* as potential candidate biomarkers for DCM, providing a new theoretical foundation and hypotheses for future mechanistic and translational research.

## 1 Introduction

Dilated cardiomyopathy (DCM) is a primary myocardial disease with unknown aetiology, characterized by ventricular dilation accompanied by impaired systolic function [1]. Clinical manifestations include decreased cardiac function, often accompanied by arrhythmias, which significantly reduce patients' quality of life and shorten life expectancy [2]. Studies indicate that approximately 1 in 250 individuals worldwide develops DCM, accounting for 40% of heart failure cases [1, 2]. Although drugs such as SGLT2 inhibitors and β‐receptor blockers are used clinically, therapeutic outcomes remain unsatisfactory due to the complexity of DCM pathogenesis and a lack of key intervention targets [3, 4]. Thus, it is of great significance to identify candidate biomarkers that may facilitate the early detection of DCM and provide potential clues for disease prevention or intervention.

Normally, the myocardium primarily relies on oxidative phosphorylation for energy production [5]. DCM patients often present with energy metabolism disorders and mitochondrial dysfunction, leading to reduced oxidative phosphorylation, insufficient ATP production, and failure to meet normal myocardial demand [6, 7]. Consequently, metabolic reprogramming occurs in DCM cardiomyocytes, where anaerobic glycolysis becomes the primary source of ATP, and lactate is the main product of anaerobic glycolysis [8, 9]. Emerging evidence demonstrates that lactate is not merely a metabolic byproduct, but also an important signalling molecule involved in immune regulation, inflammatory responses and cardiovascular remodelling [10]. Lactylation is a recently identified post‐translational modification driven by intracellular lactate accumulation. Under conditions of enhanced glycolysis, increased lactate can be converted into lactyl‐CoA, which serves as a donor for lysine lactylation on histones or non‐histone proteins. Histone lactylation regulates chromatin accessibility and transcriptional activity, thereby influencing the expression of genes related to inflammation, fibrosis, apoptosis and metabolic remodelling [11, 12, 13]. In cardiovascular disease, excessive lactate has been implicated in pathological remodelling processes through lactylation‐dependent regulatory mechanisms [14, 15]. Specifically, lactate accumulation induces BRD4 lactylation, which subsequently activates the NF‐κB signalling pathway, promotes the release of pro‐inflammatory cytokines, and contributes to myocardial fibrosis in heart failure [15, 16]. In hypertrophic cardiomyopathy, lactate activates the ERK pathway by stabilizing NDRG3, promoting gene expression associated with myocardial hypertrophy [17, 18]. These findings suggest that lactylation‐related genes may serve as candidate biomarkers in the cardiovascular system; however, the lactylation‐related genes in DCM remain largely unclear.

**FIGURE 1:**
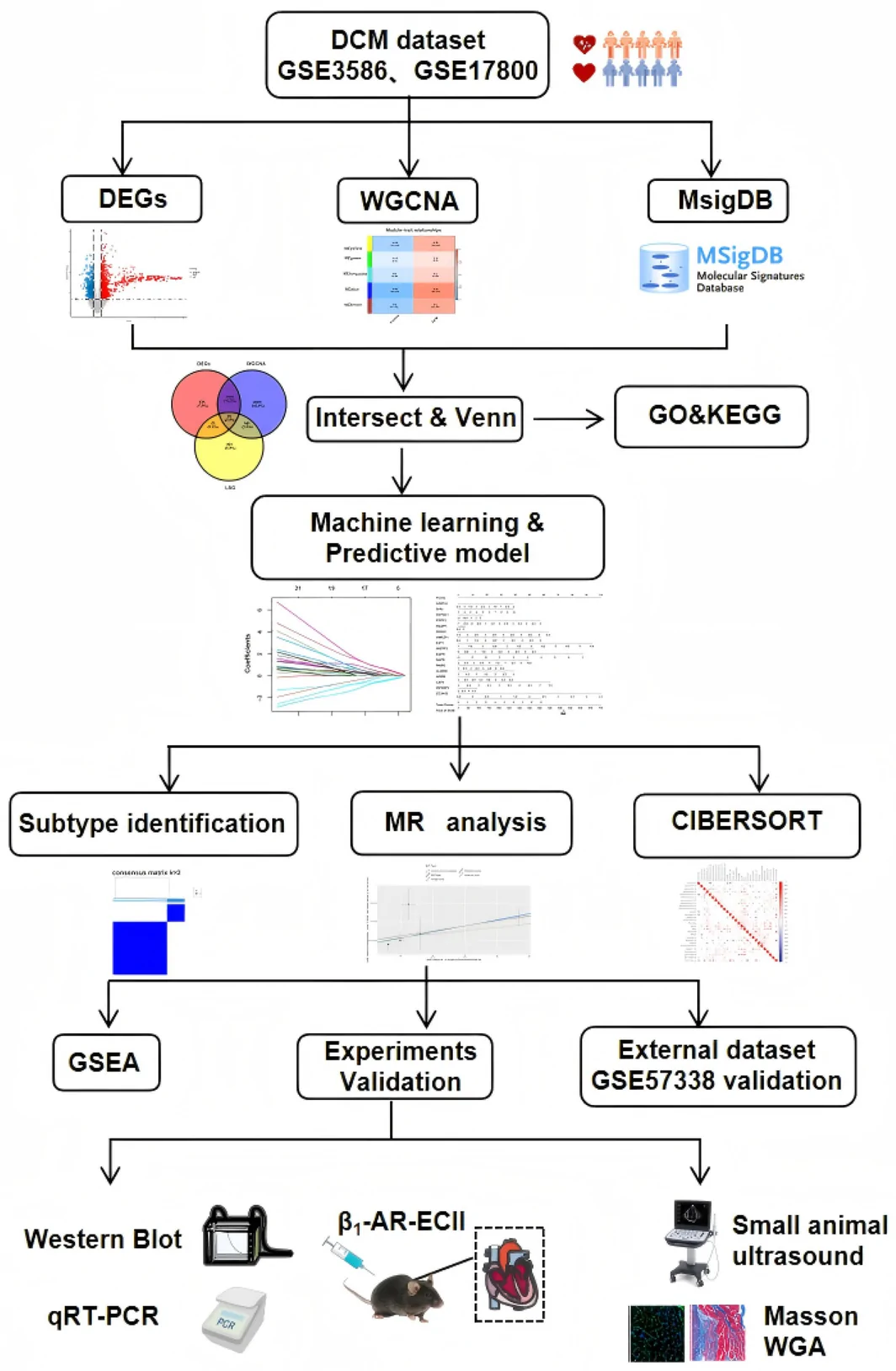
The flowchart of this study.

To address this gap, we obtained DCM‐related datasets from the GEO database, screened for differentially expressed genes between controls and DCM patients, and integrated WGCNA with lactylation‐related genes (LRGs), ultimately identifying 38 LRGs closely associated with DCM. Through GO and KEGG enrichment analyses, the biological processes and signal pathways involved in these genes were systematically evaluated. Further, 18 key LRGs were screened by a machine learning algorithm. Subsequently, we employed the CIBERSORT algorithm to analyse the immune infiltration of DCM, revealing significant dysregulation of inflammatory responses, particularly involving CD8^+^ T cells, CD4^+^ T cells, neutrophils, and natural killer T cells. Notably, the expression of the 18 key LRGs was strongly correlated with these immune cell subsets. Based on their expression patterns, DCM patients were divided into two subtypes via consensus clustering, and their differences were compared. Mendelian randomization suggested potential causal associations of *EXT1* with heart failure and *IER3* with DCM. Candidate genes were further validated using an external dataset and a β_1_‐AA‐induced heart failure model with DCM characteristics. The hub genes were further confirmed by RT‐PCR and Western blot. A detailed flowchart of the analysis is presented in Figure 1. This study provides a new theoretical basis and research direction for exploring the mechanisms of DCM.

## 2 Results

### 2.1 Identification of Differentially Expressed Genes (DEGs) in DCM

**FIGURE 2:**
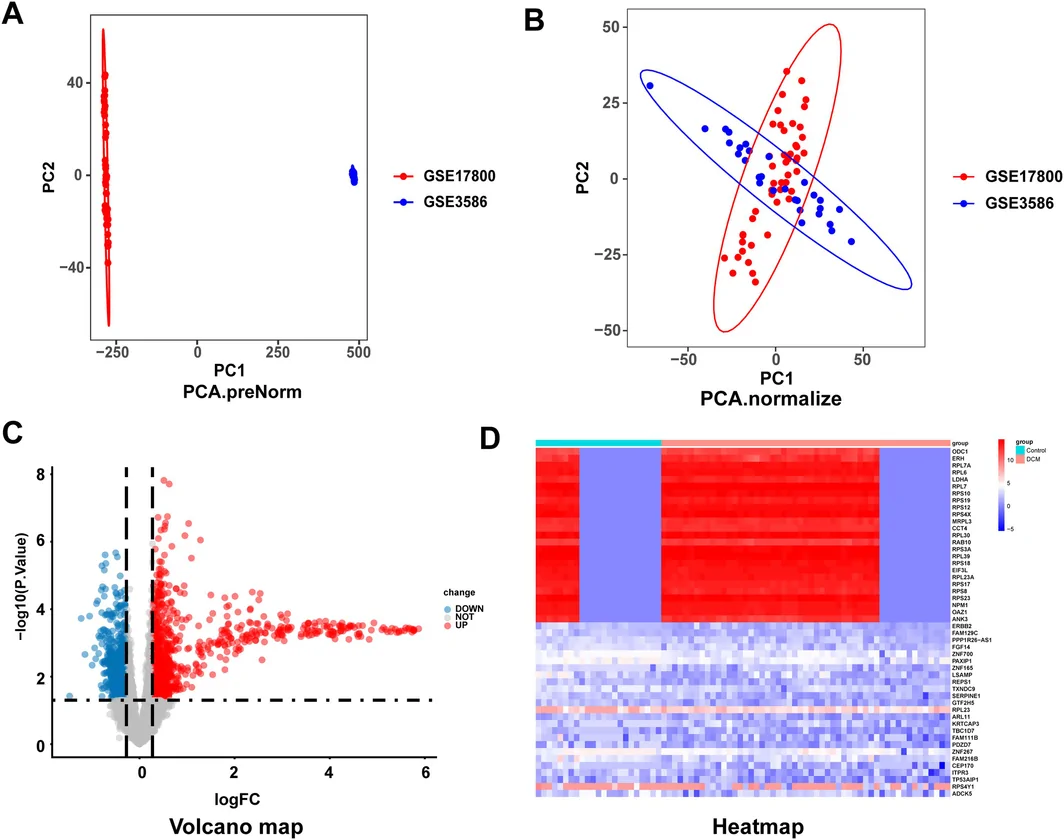
Screening of differentially expressed genes. (A) PCA before batch effect removal. (B) PCA after batch effect removal. (C) Volcano plot of DEGs. (D) Heatmap of DEGs.

This study integrated two microarray datasets, GSE3586 and GSE17800, comprising a total of 76 myocardial tissue samples, including 23 normal controls and 53 DCM samples, covering 37,259 genes. Batch effects were corrected and expression data were standardized to ensure accuracy and consistency of subsequent analyses (Figures S2A and S2B). Principal component analysis (PCA) showed improved clustering after batch effect correction (Figure 2A,B), indicating effective suppression of batch effects. Differential expression analysis identified 1716 DEGs (|log_2_FC| ≥ 0.58, *p* < 0.05), including 1117 upregulated and 599 downregulated genes. The distribution of these DEGs is visualized in volcano and heatmap plots (Figure 2C,D), highlighting significant differences between DCM and control groups.

### 2.2 Identification of Gene Modules Related to DCM by WGCNA and Screening of Lactylation‐Related Genes

**FIGURE 3:**
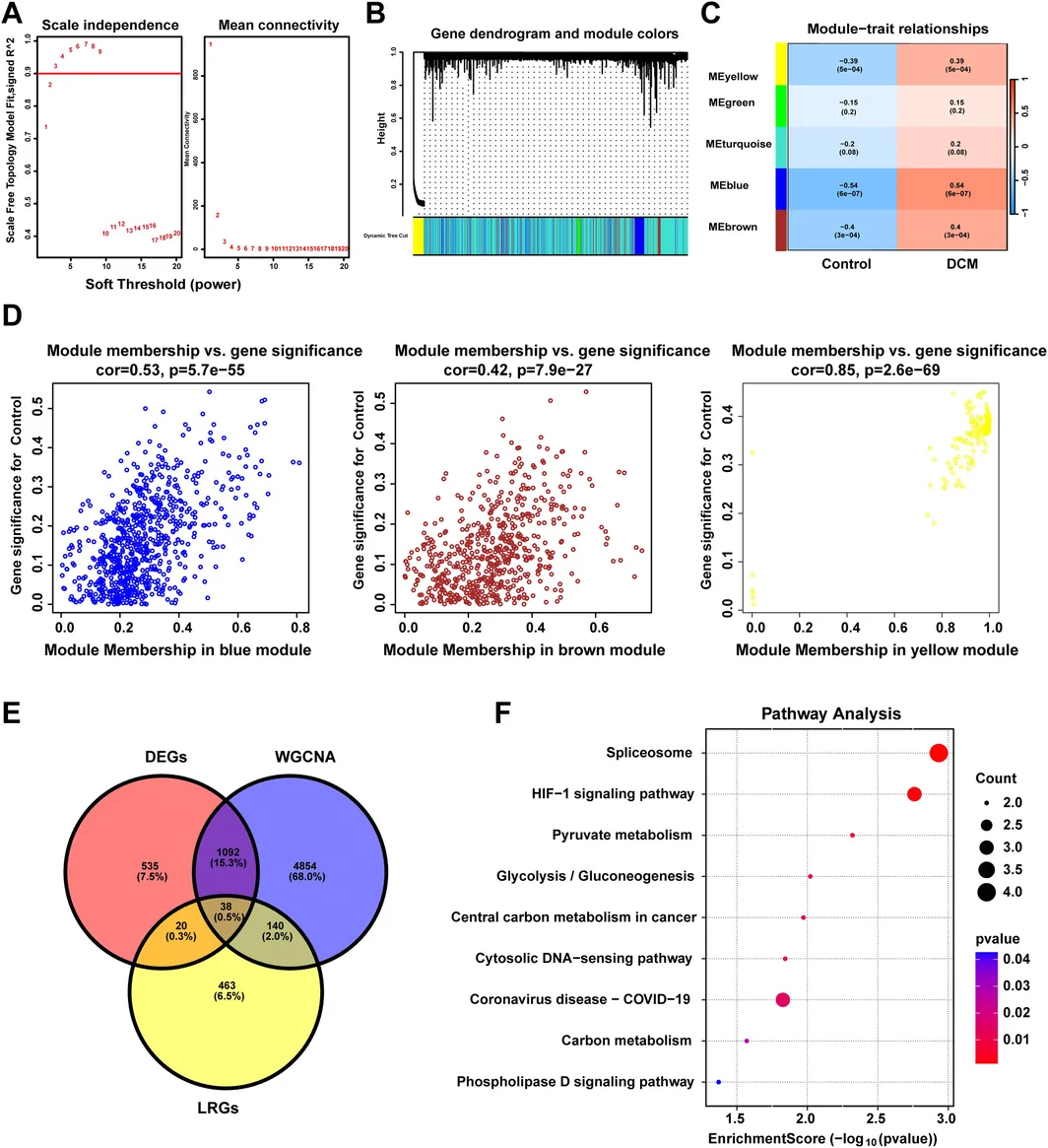
Identification of gene modules associated with DCM by WGCNA and screening lactylation‐related genes. (A) Analysis of the scale‐free fit index (left) and mean connectivity (right) for different soft‐thresholding powers. (B) Gene dendrogram and modules. (C) The correlation heatmap between gene modules and DCM status. (D) Scatter plots showing the correlation between module membership (MM) and gene significance (GS) in the yellow, blue, and brown modules. (E) Venn diagram showing the intersection of DEGs, WGCNA, and LRGs. (F) KEGG enrichment analysis.

Weighted gene co‐expression network analysis was performed to identify DCM‐related modules. In this study, the power parameters were calculated to ensure that the network conforms to the scale‐free characteristics, the power range was from 1 to 20, and the scale‐free *R* ^2^ = 0.9 was used as the fitting standard of the scale‐free network. When the soft threshold was 2.3, *R* ^2^ reached 0.9 for the first time, indicating that the network structure was close to a scale‐free distribution (Figure 3A). Then, dynamic tree cutting was performed, followed by hierarchical clustering analysis, which identified multiple co‐expressed gene modules (Figure S3A–C). In the identification of gene modules, five modules were finally identified and marked with different colours. By calculating the gene significance of each module, the modules with significance level *p* < 0.05 were considered to be closely related to DCM (Figure 3B,C). Finally, correlation analysis between modules and clinical traits showed that the yellow (*r* = 0.85, *p* = 2.6e−29), blue (*r* = 0.53, *p* = 5.7e−55), and brown (*r* = 0.42, *p* = 7.9e−27) modules were significantly positively correlated with the DCM group and negatively correlated with controls (Figure 3D), suggesting that these modules may be involved in DCM pathogenesis.

Lactylation‐related data were downloaded from the MisGDB database, and intersection with the DEGs and WGCNA module genes yielded 38 core genes as shown in the Venn diagram (Figure 3E). GO and KEGG enrichment analyses revealed that these genes were enriched in biological processes related to RNA stability and metabolic regulation (Figure S3D), cellular components enriched in spliceosome formation (Figure S3E), molecular functions enriched in protein binding (Figure S3F), and pathways including pyruvate metabolism and glycolysis/gluconeogenesis metabolism (Figure 3F).

### 2.3 Machine Learning Algorithm to Screen Key Genes With Diagnostic Value

**FIGURE 4:**
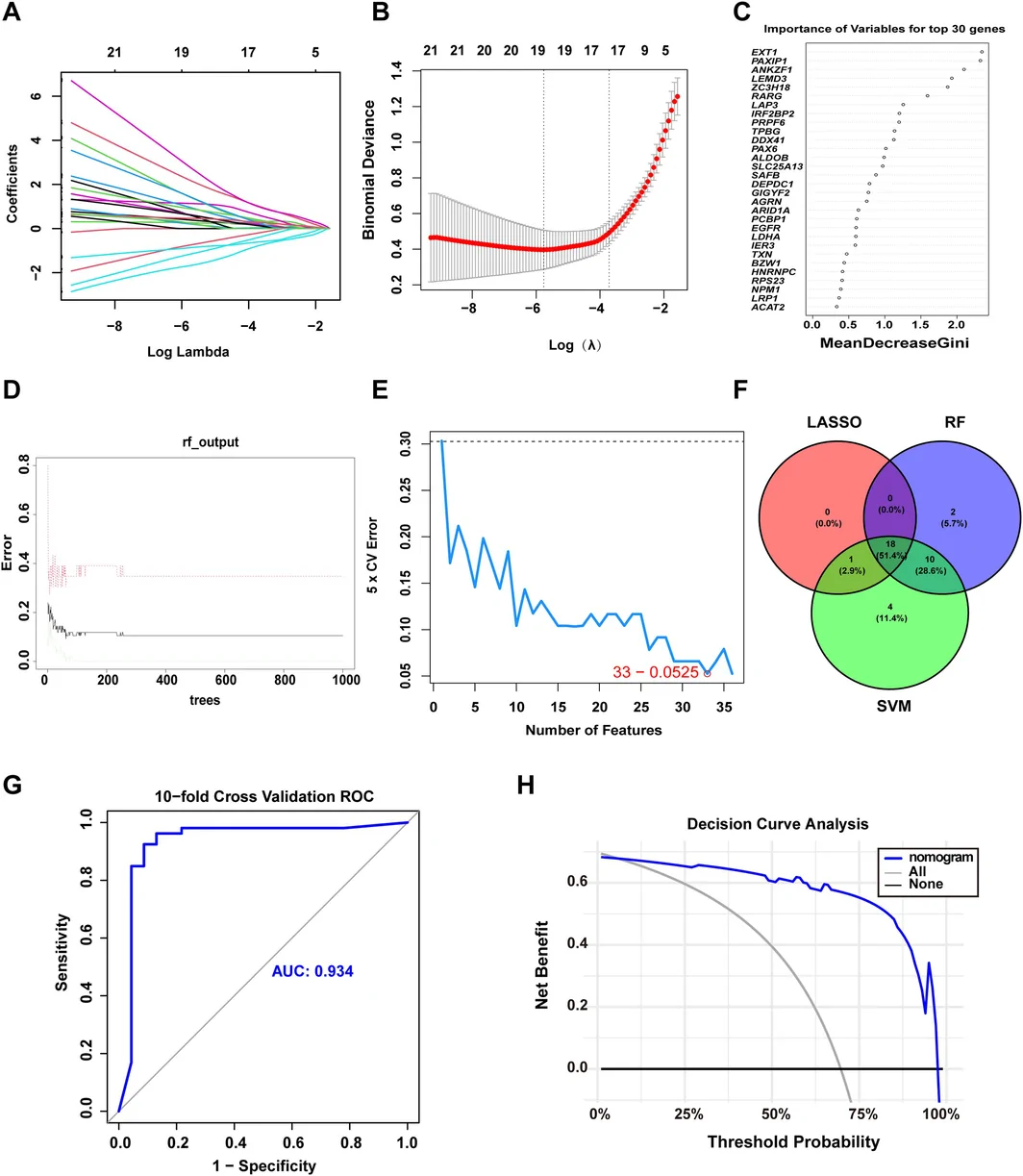
Machine learning screening of key genes and construction of risk prediction Model. (A) LASSO coefficient distribution plot for cross genes. (B) Cross‐validation for selecting the optimal tuning parameter log(*λ*) in LASSO regression analysis. (C) Gene feature importance score plot for the RF model. (D) Training error plot for the RF model. (E) Relationship between Error Rate and Number of Features in the SVM model during Cross‐Validation. (F) Venn diagram of genes from three algorithms. (G) ROC curve of the nomogram. (H) Decision curve analysis plot.

LASSO, random forest (RF), and support vector machine (SVM) were used to assess the importance of the 38 genes. In the LASSO coefficient distribution chart, the lower horizontal axis was the logarithm of *λ*, the upper horizontal axis was the number of variables, the vertical axis represents the numerical value of the coefficient, and different lines represent different variables. With the increase of *λ*, the complexity of the model decreases, the coefficients approach 0, and variables compressed to 0 later were more important (Figure 4A). Cross‐validation was then performed. The lower *x*‐axis was the logarithm of *λ*, the upper *x*‐axis was the number of variables, and the *y*‐axis was the binomial deviation, reflecting the model's fit (smaller values indicate better fit). Therefore, the *λ* value and the corresponding number of variables at the left dotted line in the plot were selected (Figure 4B). Ultimately, the LASSO regression model screened 19 important genes.

In the RF model, the top 30 genes with the highest score were selected as important genes, as shown in Figure 4C, the ordinate is the gene name, and the abscissa is MeanDecreaseGini, which indicates the impact of each variable on the heterogeneity of observations at each node of the classification tree, so it can be used to compare the importance of variables. A larger value indicates a more significant impact of the variable on classification and higher importance. The training error diagram of the model shows that with the increase in the number of trees in the model, the error on the training set gradually decreases (Figure 4D). The optimal feature set for the support vector machine (SVM) was determined through 5‐fold cross‐validation, revealing that 33 features yielded the minimum error rate of 5.25% (Figure 4E). Intersection of gene sets from the three methods yielded 18 overlapping genes: *IER3*, *ARID1A*, *DEPDC1*, *PRPF6*, *PAXIP1*, *DDX41*, *ANKZF1*, *EXT1*, *GIGYF2*, *EGFR*, *SAFB*, *RARG*, *ALDOB*, *AGRN*, *LAP3*, *IRF2BP2*, *ZC3H18*, and *TPBG* (Figure 4F).

Based on these 18 key genes, a risk prediction nomogram for DCM was constructed using the “rms” package. In the nomogram, each gene corresponds to a score, and the total score of these genes was used to predict the risk of DCM (Figure S4A). The ROC curve showed that the AUC of the model reached 0.934 (Figure 4G), indicating high predictive accuracy. The calibration curve was highly coincident with the ideal curve (Figure S4B), which showed that the corrected risk prediction model had a good prediction effect on DCM risk. The decision curve analysis demonstrated net benefit across a threshold probability range of 0.2–0.8 (Figure 4H), supporting the model's clinical utility.

### 2.4 Immune Infiltration Analysis Showed That Hub Genes Significantly Correlated With Immune Infiltration

**FIGURE 5:**
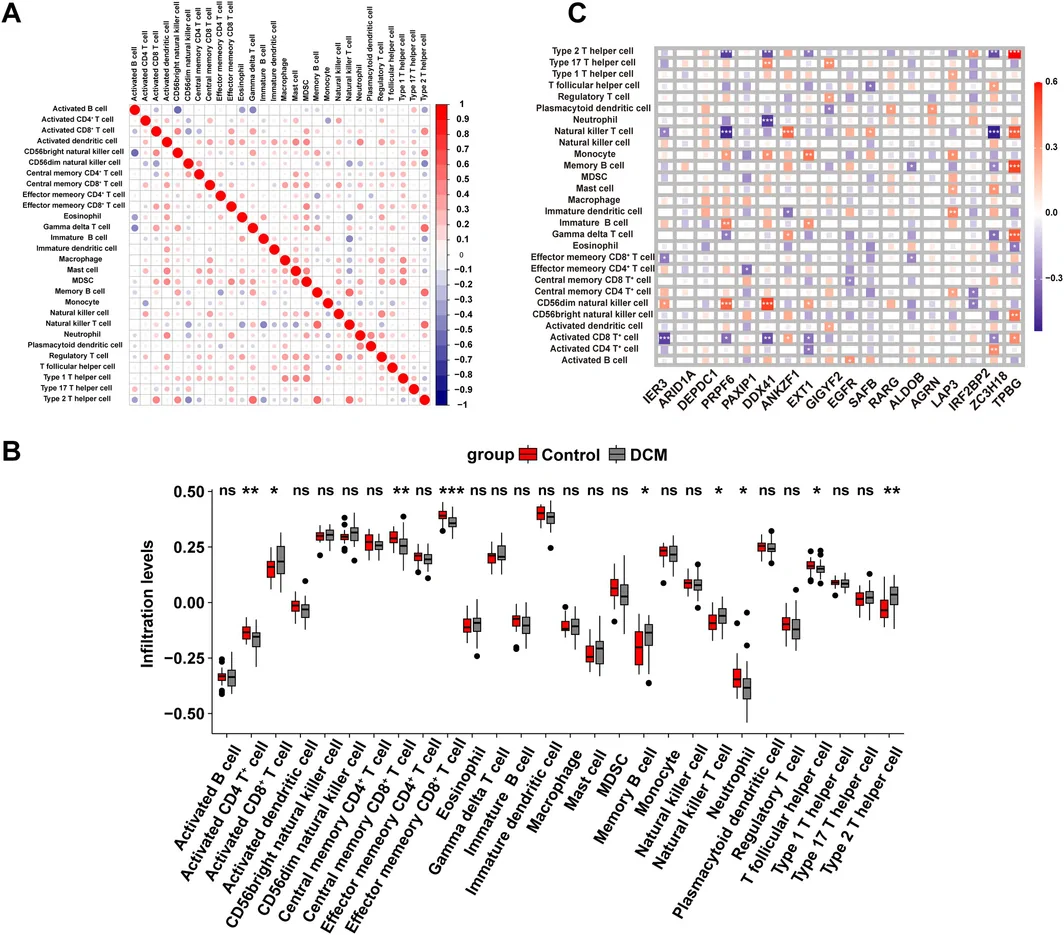
Correlation analysis of immune infiltration. (A) Correlation heatmap of immune scores. (B) Comparison of immune cell infiltration differences. (C) Correlation analysis between the 18 screened genes and immune cells. ***0 < *p* < 0.001, **0.001 < *p* < 0.01, *0.01 < *p* < 0.05, ns (not significant): 0.05 < *p* < 1.

Based on the analysis dataset, the CIBERSORT algorithm was used to analyse the composition of immune infiltrating cells. The results showed that compared with the normal group, there were significant differences in various immune cell types in DCM patients, including CD4^+^ T cells, neutrophils, natural killer T cells, and CD8^+^ T cells (Figure 5A,B). The correlation between 18 hub genes and 22 kinds of immune cell infiltration was further studied. As shown in Figure 5C, CD4^+^ T cells positively correlated with *ZC3H18* expression, while neutrophils negatively correlated with *DDX41* expression. These results indicated that immune cell infiltration was significantly altered in DCM and the hub genes correlated with immune cell infiltration.

### 2.5 Subtype Construction and Functional Analysis Based on Hub Gene

**FIGURE 6:**
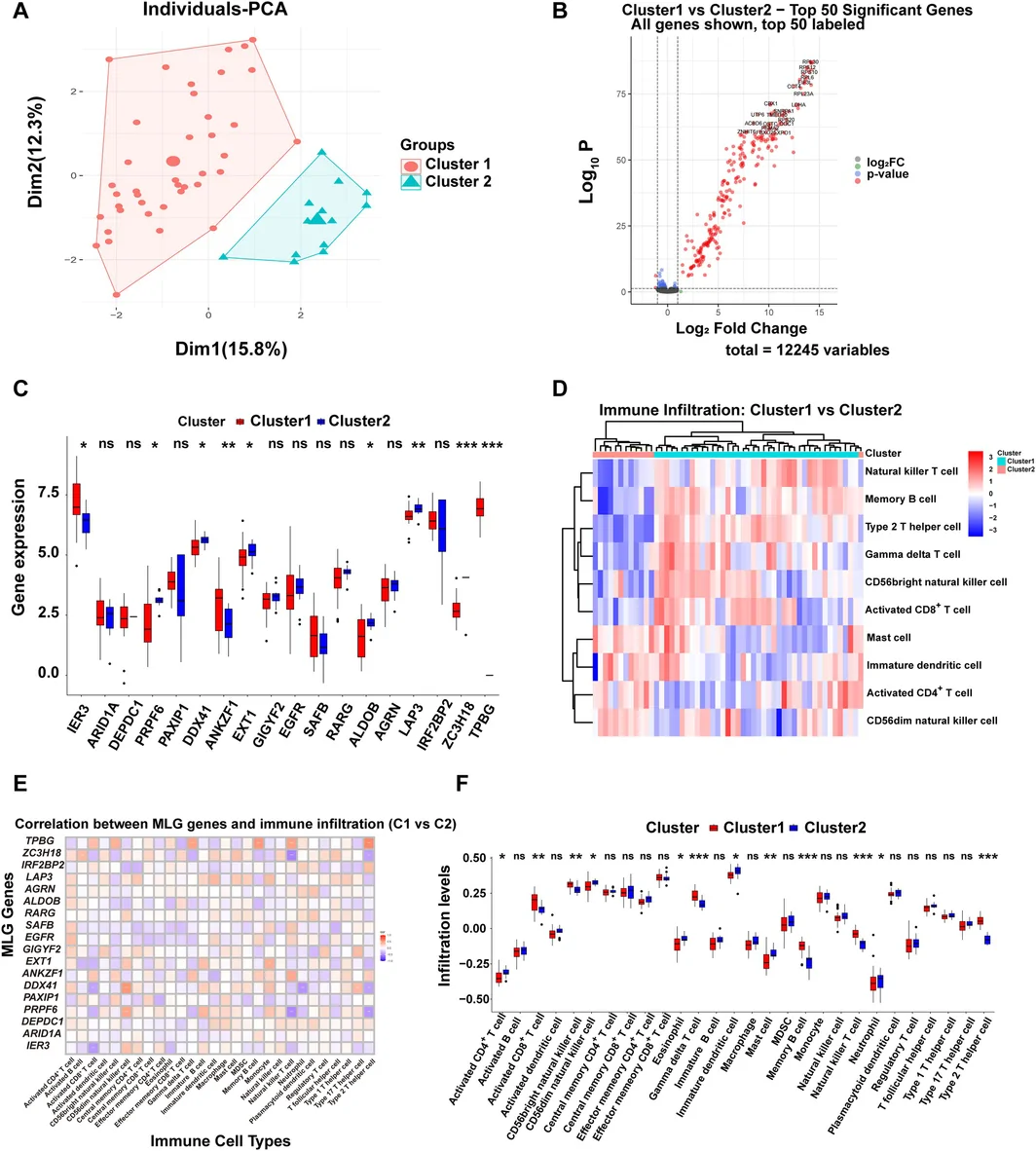
Sample cluster classification. (A) PCA scatter plot coloured by 2 clusters (*k* = 2). (B) Top50 genes in different subtypes. (C) Box‐chart diagram comparing key genes' expression across subtypes. (D) Heatmap between different subtypes and immune cells. (E) Correlation between subtype‐related genes and immune cells. (F) Differences in immune cell distribution between subtypes. ***0 < *p* < 0.001, **0.001 < *p* < 0.01, *0.01 < *p* < 0.05, ns (not significant): 0.05 < *p* < 1.

The results of consistent cluster analysis show that when the number of clusters *k* = 2, the consensus cluster matrix shows the most obvious degree of discrimination, indicating that the samples can be divided into two distinct subtypes (Figure S5A–G). Moreover, the stability of the clustering reached its optimal level when *k* = 2, meaning this classification method is more reliable compared to other cluster numbers (Figure S5H). The PCA scatter plot showed significant differences between the two subtypes, further supporting the decision to divide the samples into two subtypes (Figure 6A).

Therefore, 53 DCM samples were divided into two subtypes: C1 (*n* = 39) and C2 (*n* = 14). There were obvious significant gene expression differences between the two subtypes. Volcano diagram showed Top50 gene between the two subtypes (Figure 6B), and box diagram showed the differential expression of 18 key genes across different subtypes. Among them, *PRPF6*, *DDX41*, *EXT1*, *ALDOB*, and *LAP3* were increased in C2 subtype, while *IER3*, *ANKZF1*, *ZC3H18*, and *TPBG* were increased in C1 subtype (Figure 6C). Subsequently, the immune infiltration among different subtypes was compared. The C1 subtype mainly activated CD8^+^ T cells, Natural killer T cell, and Type 2 helper cells, whereas C2 subtype primarily activated CD4^+^ T cells, neutrophils, CD56dim natural killer cell, and Eosinophil (Figure 6D–F). The above results suggested that different subtypes of DCM may be targeted by different molecular and immune cell interventions.

This provides important clues for a deeper understanding of the heterogeneity among DCM individuals. By dividing DCM into different subtypes, the diversity of the disease can be better understood, potentially providing a basis for personalized treatment and precision medicine. The identification of these molecular subtypes not only helps reveal the pathogenesis of DCM but also provides a theoretical basis for developing treatment strategies tailored to different subtypes. In the future, personalized treatment for different subtypes is expected to improve treatment efficacy and patient quality of life.

### 2.6 Mendelian Randomization Analysis Identifies Potential Associations of *EXT1* With Heart Failure and *IER3* With DCM

**FIGURE 7:**
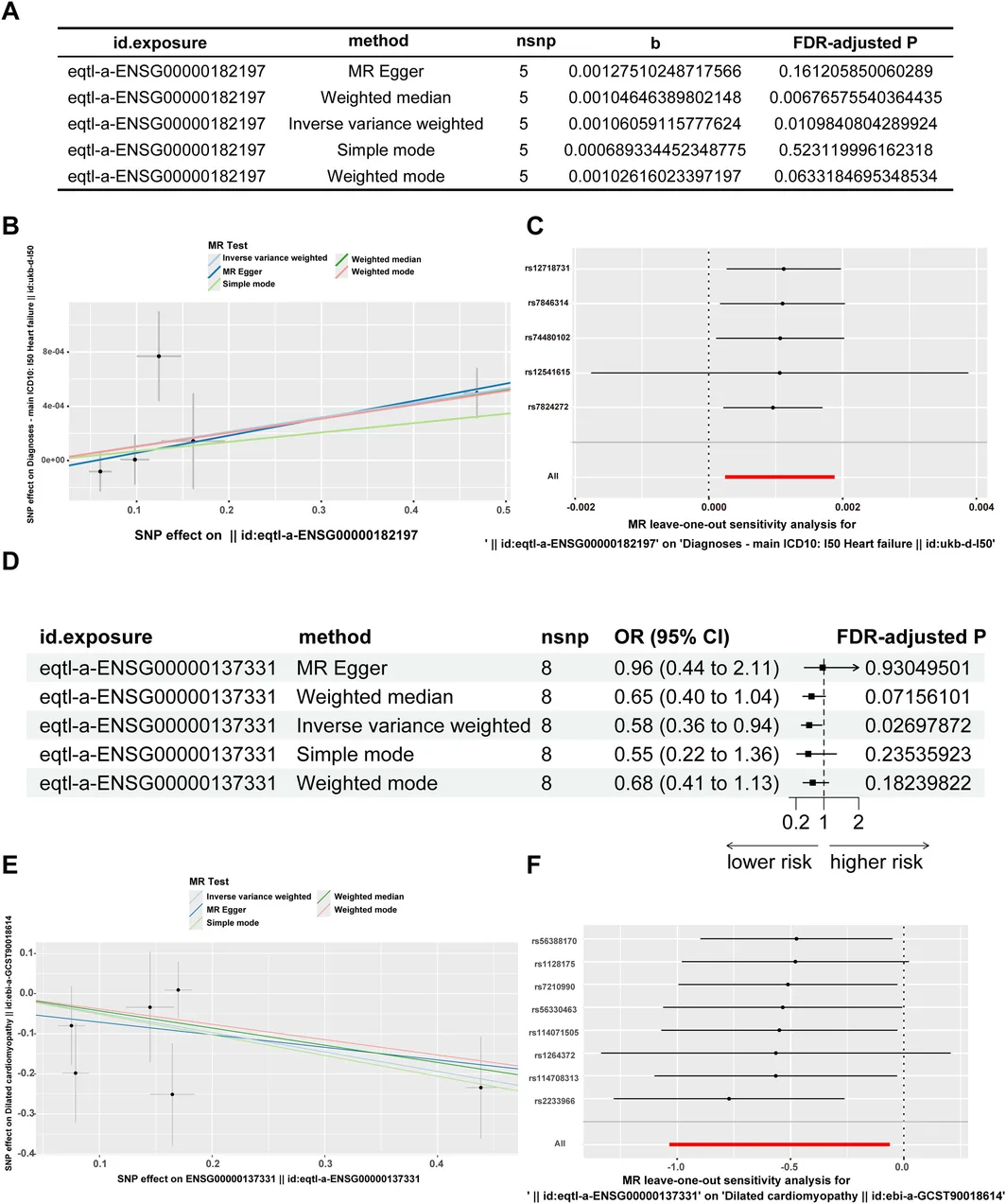
Mendel randomized analysis of *EXT1* and *IER3*. (A) The forest plot of the causal relationship between *EXT1* and heart failure using different MR methods. (B) The scatter plot showed the causal effect of *EXT1* on heart failure. (C) Leave‐one‐out sensitivity analysis for *EXT1*. (D) Forest plot of the causal relationship between *IER3* and DCM. (E) The scatter plot showed the causal effect of *IER3* on DCM. (F) Leave‐one‐out sensitivity analysis for *IER3*.

We performed two‐sample Mendelian randomization (MR) analyses using gene expression‐associated eQTLs as instrumental variables. Among the 18 candidate genes, *LAP3*, *PRPF6*, *ARID1A*, *IER3*, *TPBG*, *IRF2BP2*, and *EXT1* were retained for the heart failure analysis, whereas *LAP3* and *IER3* were retained for the DCM analysis. Among these genes, the IVW method showed that genetically predicted *EXT1* was positively associated with heart failure risk (OR = 1.0011, 95% CI: 1.0002–1.0019, FDR‐adjusted *p* = 0.011), whereas *IER3* was negatively associated with DCM risk (OR = 0.58, 95% CI: 0.36–0.94, FDR‐adjusted *p* = 0.027). To further evaluate the robustness of these associations, five MR methods were applied, including IVW, MR‐Egger regression, weighted median, weighted mode, and simple mode. For *EXT1*, the MR estimates showed a positive association with heart failure risk (Figure 7A,B). For *IER3*, the MR estimates showed a negative association with DCM risk, with a generally consistent direction across different MR methods (Figure 7D,E). These findings suggest that *EXT1* and *IER3* may be involved in heart failure and DCM, respectively.

Sensitivity analyses were further performed to assess the reliability of the MR results. Cochran's *Q* test showed no significant evidence of heterogeneity, while the MR‐Egger intercept and MR‐PRESSO global tests did not detect significant horizontal pleiotropy (Table S2). In addition, leave‐one‐out analysis showed that no single SNP substantially influenced the overall MR estimates for *EXT1* or *IER3*, supporting the stability of the results (Figure 7C,F).

### 2.7 Biological Functions and Signal Transduction Pathways of Two Characteristic Genes

**FIGURE 8:**
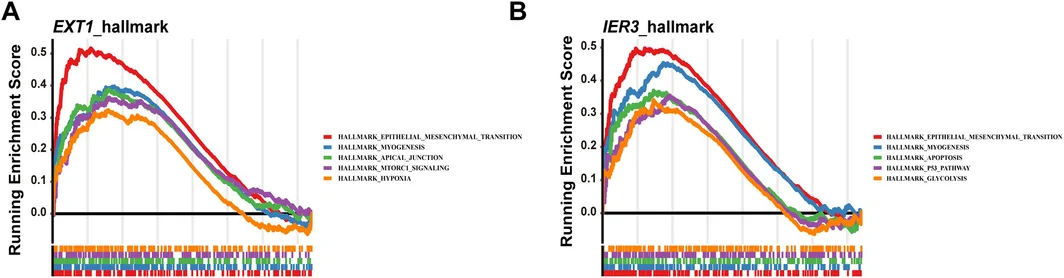
Gene set enrichment analysis (GSEA) for *EXT1* and *IER3*. (A) GSEA enrichment analysis of *EXT1*. (B) GSEA enrichment analysis of *IER3*.

Single gene GSEA was performed to elucidate the biological functions and pathways associated with *EXT1* and *IER3*. The top 5 enriched terms for each gene were shown. We found that the characteristic genes were involved in biological processes such as cell death, stress response, tissue repair, glycolysis, immune and inflammatory responses, as well as signalling pathways including p53 signal pathway, HIF‐1, and mTORC1 (Figure 8A,B). The results suggest that *EXT1* and *IER3* may play an important role in cell survival, immune infiltration, and metabolic regulation.

### 2.8 External Dataset Validation and Expression Analysis of the 2 Hub Genes

Expression and prediction performance of *EXT1* and *IER3* were further validated in the external dataset GSE57338. Consistent with MR results, *EXT1* was significantly upregulated and *IER3* downregulated in the verification set (Figure S6A). In GSE57338, the AUC values of the ROC curve were 0.880 and 0.771 for *EXT1* and *IER3*, respectively (Figure S6B). Decision curves deviated from reference lines, supporting model feasibility (Figure S6C), and calibration curves confirmed strong predictive ability (Figure S6D), suggesting that the two genes also had ideal predictive accuracy in the external validation set.

**FIGURE 9:**
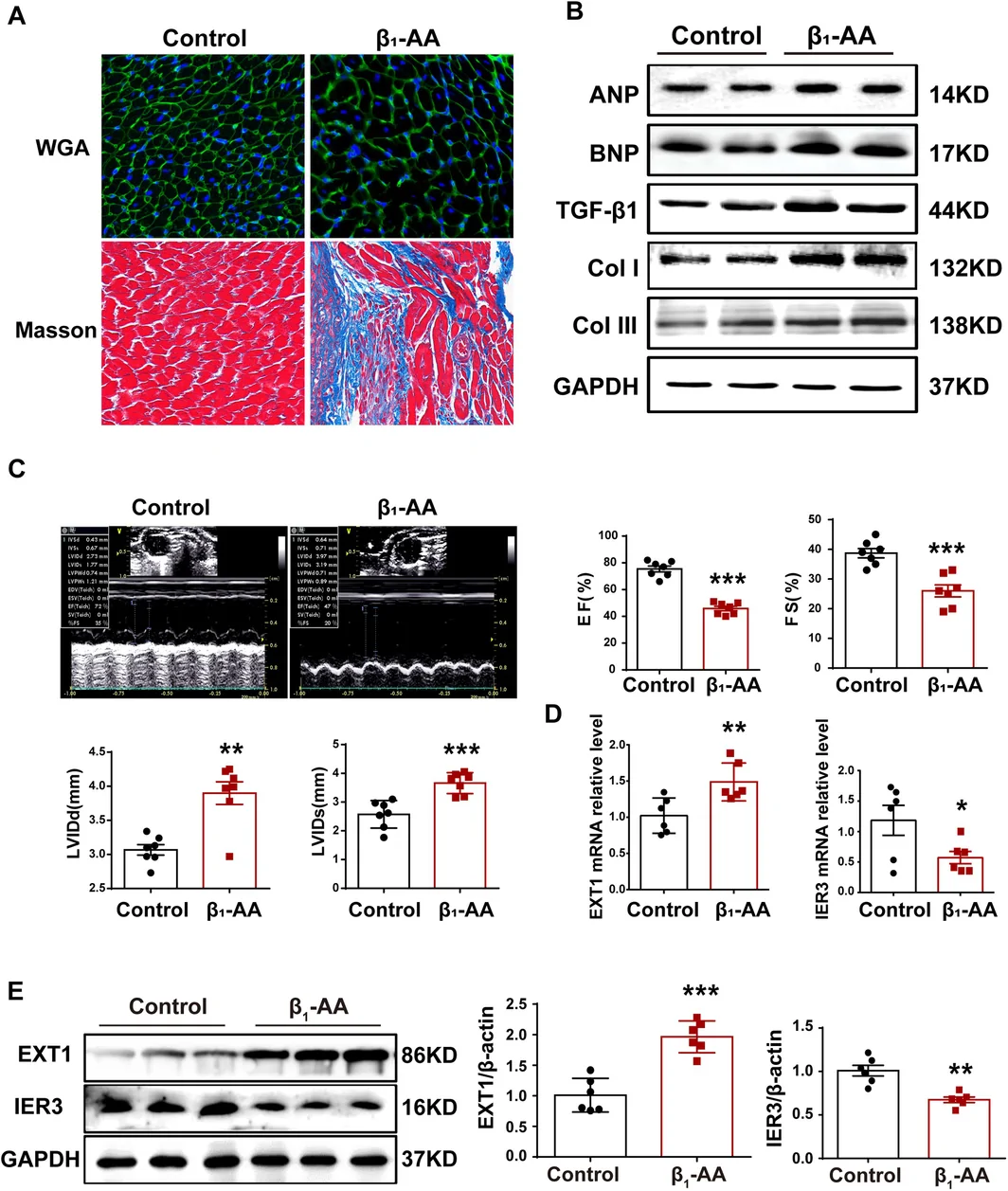
External validation and expression analysis of *EXT1* and *IER3*. (A) Masson's trichrome and WGA staining. (B) ANP, BNP, TGF‐β1, Collagen I, and Collagen III expression level (*n* = 6). (C) Cardiac function was examined by echocardiography (*n* = 7). (D) EXT1 and IER3 mRNA expression level (*n* = 6). (E) Western blot was used to detect the protein level of EXT1 and IER3 (*n* = 6). Data are presented as mean ± SEM. Each data point represents an individual biological replicate *p < 0.05, **p < 0.01, ***p < 0.001.

We have been committed to the study of β_1_‐AA (β_1_‐adrenergic receptor autoantibodies) causing heart failure, which could be detected in the serum of 40%–60% patients with heart failure. Removal of serum β_1_‐AA by immunoadsorption significantly improves cardiac function of patients. Given that DCM is one of the major causes of heart failure, we further established a β_1_‐AA induced heart failure model to evaluate whether the identified hub genes were associated with DCM‐related pathological remodelling. Compared with the control group, Masson's trichrome staining revealed a significant increase in interstitial collagen deposition in the model group, while WGA staining demonstrated a marked enlargement of the cardiomyocyte cross‐sectional area (Figure 9A), indicating typical myocardial fibrosis and hypertrophy. Consistent with the phenotypic changes, *ANP* and *BNP* were significantly upregulated in the heart of the model group. In addition, the fibrosis‐related markers *TGF‐β1*, *Collagen I*, and *Collagen III* were also markedly elevated (Figures 9B and S6E). Echocardiographic analysis showed that the model group exhibited a significant decrease in left ventricular ejection fraction (EF%) and fractional shortening (FS%), along with a significant increase in left ventricular internal diameter at end‐diastole (LVIDd), end‐systole (LVIDs) (Figure 9C). These findings indicated that the β_1_‐AA model exhibits key DCM‐like pathological features. RT‐PCR and Western blot analyses showed that *EXT1* was significantly up‐regulated and *IER3* was down‐regulated in the model group (Figure 9D,E), consistent with MR and external validation results.

## 3 Discussion

Dilated cardiomyopathy (DCM) is a complex and heterogeneous myocardial disease, and its pathogenesis involves the interaction of many factors such as heredity, metabolism, and immune inflammation [1, 19]. By integrating bioinformatics, machine learning algorithm, combining Mendel randomization (MR) and experimental validation, we systematically identified lactylation‐related genes (LRGs) associated with DCM. Among these, *EXT1* showed increased expression, whereas *IER3* showed decreased expression in both the external validation dataset and the β_1_‐AA‐induced heart failure model with DCM‐like pathological features. Collectively, these findings support the potential of *EXT1* and *IER3* as candidate biomarkers for DCM and provide novel insights into the possible involvement of lactylation‐related molecular mechanisms in DCM pathogenesis.

In recent years, the role of metabolic reprogramming in cardiovascular diseases has attracted increasing attention [20]. The myocardial energy metabolism of DCM patients is disordered, which is characterized by impaired oxidative phosphorylation and enhanced glycolysis, leading to lactate accumulation [7]. Lactate is not only a byproduct of energy metabolism, but also can regulate gene expression through histone lactylation and participate in inflammation and immune response [11, 15]. Mechanistically, lactylation provides a molecular link between metabolic reprogramming and transcriptional regulation [11, 21]. In DCM, impaired mitochondrial oxidative phosphorylation and compensatory enhancement of glycolysis may increase intracellular lactate levels [7, 22]. Elevated lactate can be converted to lactyl‐CoA, which serves as a substrate for acyltransferases such as p300 and KAT2A. These enzymes catalyse the transfer of lactyl groups to lysine residues on histones, thereby altering transcription of genes involved in inflammatory activation, extracellular matrix remodelling, oxidative stress, and cardiomyocyte survival [11, 23, 24]. Consistent with this, H4K12 lactylation potentiates mitochondrial oxidative stress via the Foxo1 pathway [25]. Research has also indicated that lactylation of Snail1 exacerbates cardiac dysfunction by promoting endothelial‐to‐mesenchymal transition (EndoMT) through activation of the TGF‐β/Smad2 pathway [26]. However, the lactylation‐related genes in DCM remain unclear. In this study, 38 LRGs related to DCM were identified by differential expression analysis and WGCNA module screening. Enrichment analysis showed that these genes were mainly involved in pyruvate metabolism, glycolysis/gluconeogenesis and other pathways, which further supported the key role of lactate metabolism in the pathogenesis of DCM. In addition, the analysis of immune infiltration showed that the CD4^+^ T cells, CD8^+^ T cells, natural killer T cell and neutrophils in DCM patients were significantly correlated with key LRGs (such as *ZC3H18* and *DDX41*), suggesting that lactylation may promote the progress of DCM by regulating the immune microenvironment. In the future, we can analyse how lactylation affects the distribution and function of myocardial immune cell subsets through single cell sequencing or spatial transcriptome technology.

In this study, three machine learning algorithms, LASSO, Random Forest (RF), and Support Vector Machine (SVM), were used to screen out 18 DCM‐related LRGs, and a high‐precision (AUC = 0.934) risk prediction model was constructed. According to consistent cluster analysis, DCM patients were divided into two subtypes (C1 and C2) with distinct gene expression patterns; *IER3*, *ANKZF1*, *ZC3H18*, and *TPBG* showed higher in the C1 subtype, whereas *PRPF6*, *DDX41*, *EXT1*, and *ALDOB* showed higher in the C2 subtype. The distribution of immune cells was different among different subtypes. Subtype C1 mainly activated CD8^+^ T cells, gamma delta T cells, memory B cells, natural killer T cells, and Type 2 T helper cells, while subtype C2 mainly activated CD4^+^ T cells, CD56dim natural killer cells, and neutrophils. These findings highlight DCM heterogeneity and provide a basis for developing subtype‐specific treatment strategies. For example, C1 subtype with high expression of *IER3* may benefit from drugs targeting CD8^+^ T cells and memory B cells, whereas C2 subtype with high expression of *EXT1* may require agents targeting CD4^+^ T cells and neutrophils.

Mendelian randomization analysis further highlighted *EXT1* and *IER3* as candidate genes with potential relevance to DCM‐related pathological processes. *EXT1*(Exostosin‐1) was identified as a risk factor for heart failure (OR = 1.0011, *p* = 0.011). *EXT1* encodes a glycosyltransferase located in endoplasmic reticulum, which forms a complex with *EXT2* to participate in the synthesis of heparan sulfate (HSPG) [27]. HSPG plays a key role in the remodelling of extracellular matrix (ECM) and the regulation of growth factor signals (such as FGF and WNT) [28]. Studies show that *EXT1* is elevated in lung adenocarcinoma, activating glycolysis and increasing lactate production and lactylation, associated with poor prognosis and metastasis in patients [29]. Recent research found that downregulation of *EXT1* diminished endothelial glycocalyx, which leads to the impairment of vasomotor properties in atherosclerosis [30]. In the myocardial infarction model, HSPG inhibited cardiomyocyte repair via FGF2 [31]. Research showed that *EXT1* is significantly elevated in patients with heart failure and may serve as a diagnostic marker [32]. Our data suggest that *EXT1* may participate in DCM development through mTORC1 signal pathway.

Mendelian randomization analysis showed that *IER3* (*Immediate* Early Response 3) was a protective factor for DCM (OR = 0.58, *p* = 0.027). As a stress response gene, *IER3* plays an important protective role in many pathological conditions [33]. Its mechanism involves many aspects of regulation: under mechanical stress, IER3 upregulation improves bone metabolism by increasing lactate levels and interacting with lactate dehydrogenase‐B (LDHB) in bone marrow mesenchymal stem cells (BMSCs) [34]. In cardiomyocytes, *IER3* is regulated by stress signal pathways such as NF‐κB, and maintains cell survival by targeting the promoter region of the anti‐apoptosis gene [35]. Studies have shown that overexpression of *IER3* in neonatal rat cardiomyocytes significantly reduced hypoxia‐reoxygenation‐induced intracellular and mitochondrial ROS accumulation and cell apoptosis [36]. Conversely, *IER3* down‐regulation led to abnormal glycolysis and oxidative stress, forming a vicious circle that accelerated cardiac dysfunction [37]. In addition, the increased IER3 can promote the phosphorylation and nuclear translocation of PKCε, and reduce the accumulation of ROS, thus inhibiting the apoptosis and necrosis of cardiomyocytes, thereby alleviating myocardial infarction injury [36]. Notably, IER3 overexpression attenuated myocardial hypertrophy induced by mechanical tension, phenylephrine, or endothelin‐1 [38], further supporting its potential cardioprotective effect.

One limitation of this study is that the eQTL instruments were derived from whole‐blood expression data rather than cardiac tissue. Given the tissue‐specific nature of gene regulation, the identified associations may not fully reflect transcriptional effects in the myocardium. Nevertheless, whole‐blood eQTL datasets provide substantially larger sample sizes and greater statistical power than currently available cardiac tissue‐specific resources. Importantly, we further validated candidate genes' expression in myocardial tissues and observed significant dysregulation of *EXT1* and *IER3*, supporting their biological relevance to DCM. Future studies utilizing cardiac tissue‐specific eQTL datasets are needed to further confirm these findings.

We validated *EXT1* and *IER3* in the external dataset GSE57338. Compared with the control group, *EXT1* was upregulated and *IER3* was downregulated, which was consistent with Mendel analysis. The decision curve and calibration curve further supported the predictive performance of the model. Given that DCM is one of the major causes of heart failure, we further established a β_1_‐AA induced heart failure model with DCM‐like pathological features to validate the hub genes. Accordingly, in the model group, cardiac dysfunction manifested as decreased EF% and FS%, together with increased LVIDd and LVIDs. Masson's trichrome staining revealed prominent blue fibrotic deposits in both the interstitial and perivascular regions of myocardial tissues, while WGA staining indicated a significant increase in the cross‐sectional area. Furthermore, *ANP*, *BNP*, *TGF‐β1*, *collagen I*, and *collagen III* were all markedly elevated. Collectively, these results confirmed the successful establishment of the model. RT‐PCR and Western blot confirmed that compared with the control group, EXT1 was upregulated, while IER3 was downregulated in the β_1_‐AA group. GSEA analysis suggests that these genes may delay the deterioration of DCM through anti‐apoptosis or anti‐inflammatory mechanisms. These findings not only provide a new perspective for the molecular mechanism of DCM, but also suggest that *EXT1* inhibitors or *IER3* agonists may represent potential therapeutic strategies. For example, targeting the *EXT1‐HSPG* axis could reduce the infiltration of inflammatory cells, while enhancing the expression of *IER3* may protect cardiomyocytes from stress damage.

Although this study revealed the role of LRGs in DCM through multi‐omics methods, there are still some limitations. First, the data sources are limited to public databases, and the sample size is small, so it is necessary to expand the clinical cohort to validate the diagnostic efficacy of key genes. The experimental verification of lactylation modification is insufficient. In the future, it is necessary to clarify the lactylation level of key genes and their functional effects by mass spectrometry or immunoprecipitation. Secondly, the specific mechanisms of *EXT1* and *IER3* are unclear, and their regulatory networks require further exploration through cell or animal models.

## 4 Materials and Methods

### 4.1 Data Acquisition

The DCM related transcription datasets were obtained from the GEO database (https://www.ncbi.nlm.nih.gov/geo/), with selection criteria included: expression profiling by array; *Homo sapiens* ; the samples are heart tissues. Finally, the GSE17800 (platform GPL570) [39] and GSE3586 (platform GPL3050) [40] were selected for training analysis. The GSE57338 (platform GPL11532) [41] was used as validation cohorts. GSE17800 contains 6 control and 42 DCM myocardial tissues; GSE3586 contains 17 control and 11 DCM myocardial tissues; GSE57338 contains 136 control and 82 DCM myocardial tissues. Lactylation‐related genes were downloaded from the MsigDB (https://www.gsea‐msigdb.org/gsea/msigdb/) [42].

### 4.2 Principal Component Analysis (PCA) and Differential Gene Acquisition

The datasets of GSE17800 and GSE3586 were preprocessed, including data cleaning and normalization, to ensure the consistency and comparability. Datasets were merged into a matrix, and the elimination effect of batch effect was evaluated by PCA using the SVA package. Differentially expressed genes (DEG) were analysed by limma package, with screening criteria of |log_2_FC| ≥ 0.58, and adjusted *p* < 0.05. The pheatmap (version 1.0.12), dplyr (version 2.4.0), ggplot2 (version 3.5.0), and ggrepel (version 0.9.4) packages were used to draw heatmaps and volcano plots for the DEGs. Differentially expressed genes were selected for subsequent analysis.

### 4.3 Weighted Gene Co‐Expression Network Analysis (WGCNA)

“WGCN” package [43] in the R language (version 1.73) was used to construct a co‐expression network for the corrected gene expression data, selecting genes with the top 50% variance. Gene modules with similar expression patterns were extracted using the dynamic tree cutting method, and the gene modules significantly correlated with DCM were obtained for subsequent analysis. The intersection of DEGs, WGCNA module genes, and LRGs, as well as the subsequent GO and KEGG enrichment analyses, were performed as follows: Supporting Information: Methods (Section 1.1).

### 4.4 Machine Learning Was Used to Screen Core Genes

LASSO regression, Random Forests (RF), and Support Vector Machines (SVM) are three machine learning algorithms widely used in variable screening. In order to screen the most predictive features and avoid overfitting, we use the Least Absolute Shrinkage and Selection Operator (LASSO) regression method [44]. LASSO compresses the coefficients of unimportant features to zero through L1 regularization, thus realizing feature selection. Specifically, we select the optimal penalty parameter *λ* (lambda) through 10‐fold cross‐validation, which corresponds to the model with the smallest cross‐validation error, and finally retain the characteristics of non‐zero coefficients for subsequent modelling. Random Forest (RF) is an algorithm based on ensemble learning, which improves the robustness of the model by constructing multiple decision trees and summarizing their prediction results [45]. Support Vector Machine (SVM) realizes classification/regression by finding the hyperplane with maximum interval [46]. We used the Radial Basis Function (RBF) as the kernel function, and the model performance was evaluated through 5‐fold cross‐validation. The “glmnet”, “randomForest” and “e1071” packages in R software were used for the respective analysis. Single‐gene GSEA of the candidate genes was performed using the Hallmark gene sets as detailed in Supporting Information: Methods (Section 1.2).

### 4.5 Mendelian Randomization Analysis

Mendelian randomization (MR) analyses were performed using the TwoSampleMR package (version 0.5.10) in R software (version 4.3.1). The overall workflow of the MR analysis is illustrated in Figure S1. Cis‐eQTL summary statistics for candidate genes were obtained from whole‐blood gene expression datasets from the eQTLGen Consortium, comprising individuals of European ancestry. GWAS summary statistics for dilated cardiomyopathy (ebi‐a‐GCST90018614) and heart failure (UB‐d‐i50) were obtained from publicly available GWAS databases. SNPs significantly associated with gene expression were selected as instrumental variables (IVs) at the genome‐wide significance threshold (*p* < 5 × 10^−8^). To ensure independence, LD clumping (*r* ^2^ < 0.001, window = 10,000 kb) was performed based on the European reference panel. Instrument strength was assessed using the *F*‐statistic (*F* = *β* ^2^/SE^2^), and SNPs with *F* > 10 were retained. Genes with at least three independent IVs were included in the subsequent MR analyses. Detailed information on the candidate genes, their eQTL IDs, SNP counts, and inclusion status for DCM and HF MR analyses is provided in Table S1. Exposure and outcome datasets were harmonized using the harmonise_data function in the TwoSampleMR package, and ambiguous palindromic SNPs were excluded. The inverse variance weighted (IVW) method was the primary analysis, with MR‐Egger regression, weighted median, weighted mode, and simple mode as complementary analyses. Sensitivity analyses included Cochran's *Q* test for heterogeneity, MR‐Egger intercept test for horizontal pleiotropy, MR‐PRESSO analysis where applicable, and leave‐one‐out analysis to evaluate the influence of individual SNPs. To account for multiple comparisons across candidate genes and outcomes, *p* values were adjusted using the false discovery rate (FDR) method, and an FDR‐adjusted *p* value < 0.05 was considered statistically significant.

### 4.6 Immune Infiltration Analysis

The “CIBERSORT”(version 1.03) was used to analyse immune cell infiltration levels between disease and the normal sample [47], the parameter “PERM” was set to 1000 and the cut‐off value *p* < 0.05. In addition, the proportion of each immune cell in the sample was calculated and visualized by bar graph. Spearman correlation between immune cells and genes was calculated using the “corrplot” package (version 0.95), and results were visualized with “ggplot2” (version 3.3.2).

### 4.7 Consensus Clustering Analysis and Differences Between Subtypes

Utilizing the obtained expression levels of LRGs associated with DCM, subtype identification among DCM patients was conducted. Unsupervised consensus clustering analysis was performed using the “ConsensusClusterPlus” package (version 1.64.0) [48]. To ensure clustering consistency, 1000 iterations were performed, with the sample consistency cutoff set at 0.8 and feature consistency cutoff at 1, utilizing “*k*‐means” as the clustering method. Differences in the immune environment were compared, and Gene Set Variation Analysis (GSVA) was conducted between the two subtypes [49].

### 4.8 Construction of β_1_‐AA Induced Heart Failure Model With DCM‐Like Pathological Features

C57BL/6 mice (6–8 weeks) were provided by the Experimental Animal Center of Shanxi Medical University. All animals were placed in suitable temperature (25°C ± 2°C) and humidity (50% ~ 60%). The heart failure model was established as previously described [50]. The β_1_‐AA group received subcutaneous multi‐point injections of the β_1_‐AR ECII antigen peptide on the back, once every 2 weeks for 4 weeks, with a dose of 0.4 μg/g per mouse each time. After immunization, mouse myocardial tissues were collected for analysis. Detailed procedures for Masson's trichrome staining, WGA staining, RT‐qPCR and Western blot are provided in Supporting Information: Methods (Sections 1.3–1.6), and the primer sequences are listed in Table S3. All animal procedures conformed to the Care and Use of Experimental Animals published by the National Institute of Health and were approved by the Ethics Committee of Shanxi Medical University.

### 4.9 Small Animal Ultrasound

Echocardiographic assessments were performed using a Vevo LAZR‐X photoacoustic imaging system (VisualSonics, Toronto, Canada) equipped with an MX550D linear‐array transducer (center frequency: 40 MHz). Mice were anaesthetised with isoflurane delivered via a small‐animal anaesthesia machine (induction at 3%–4%, maintenance at 1.5%–2% in 100% O_2_). The precordial region was shaved and chemically depilated using a commercial cream; the residual cream and loose hair were gently wiped away with wet cotton balls to expose the thoracic wall. Each animal was then placed in a supine position on a heated imaging platform, and warm ultrasound coupling gel was applied to the epilated chest. After a brief stabilization period (≈2–3 min) to allow heart rate to reach steady state, cardiac function was examined via M‐mode Teichholz ultrasound and the indicators of cardiac function of the mice were recorded. All measurements were averaged over at least three consecutive cardiac cycles. Upon completion of imaging, the gel was cleaned off, and the mice were monitored until fully recovered before being returned to their home cages.

## 5 Conclusion

In this study, lactylation modification was linked to the pathogenesis of DCM for the first time, identifying 18 key LRGs and confirming that *EXT1* and *IER3* are candidate genes for DCM. These findings not only deepen the understanding of the metabolic‐immune mechanism of DCM, but also provide new ideas for developing candidate biomarkers based on lactylation regulation. Further functional validation is required to elucidate the molecular mechanisms and therapeutic potential of these key genes, thereby facilitating their clinical translation and advancing precision medicine in DCM.

## Author Contributions

**Xiaoyan Zhi:** writing – original draft, writing – review and editing, investigation, methodology, formal analysis, data curation, validation. **Miao Zhang:** writing – original draft, data curation, methodology, software. **Luyao Xu:** data curation, visualization, writing – original draft. **Yajing Zhang:** data curation, visualization, writing – original draft. **Zhijun Zhang:** writing – review and editing, methodology, software, validation. **Xiaohui Wang:** project administration, writing – review and editing, supervision, funding acquisition, validation. **Li Wang:** supervision, validation, writing – review and editing, funding acquisition, resources, project administration.

## Funding

This work was supported by the Shanxi Province Higher Education “Billion Project” Science and Technology Guidance Project (BYJL007, SY‐BYSL‐2025008), the Basic Research Project of the Shanxi Science and Technology Department (No. 202303021221134), and Fund Program for the Scientific Activities of Selected Returned Overseas Professionals in Shanxi Province (20250021).

## Ethics Statement

The Committee for Ethics of Animal Experimentation approved the animal experiments, which followed the guidelines for animal experiments at Shanxi Medical University (Approval No. DWYG2025223).

## Consent

The authors have nothing to report.

## Conflicts of Interest

The authors declare no conflicts of interest.

## Supporting information


**Table S1:** Instrumental variables derived from eQTLs for 18 candidate genes and their inclusion in two‐sample Mendelian randomization analyses for dilated cardiomyopathy and heart failure.
**Table S2:** MR‐Egger, MR‐PRESSO and Cochran's *Q* test.
**Table S3:** Primer sequences.
**Figure S1:** The flowchart of Mendelian randomization analysis.
**Figure S2:** The Standardization of datasets GSE3586 and GSE17800.
**Figure S3:** Weighted gene co‐expression network analysis (WGCNA) and GO functional enrichment of the lactylation‐related genes.
**Figure S4:** Nomogram for predicting DCM risk and its calibration curve.
**Figure S5:** Consensus clustering analysis for determining the optimal number of clusters.
**Figure S6:** Validation of *EXT1, IER3* expression and their diagnostic performance in GSE57338 and the expression levels of *ANP*, *BNP*, *TGF‐β1*, *Collagen I*, and *Collagen III*.

## Data Availability

The data that support the findings of this study are available from the corresponding author upon reasonable request.
