# Supplementary material for: Identification of a Lactylation‐Related Gene Signature and Candidate Biomarkers for Dilated Cardiomyopathy
Source: J Cell Mol Med. 2026 Sep 25;30(18):e71379. doi: 10.1111/jcmm.71379 (PMC13614876; doi:10.1111/jcmm.71379)
Supplement: Supplementary file 1 [file JCMM-30-e71379-s001.doc]

**Supplemental materials**

1. Methods

1.1 Core Gene Identification and Functional Enrichment Analysis

By integrating the differential expression gene and WGCNA, the core genes closely related to DCM were screened out. The "ggvenn" package (version 0.1.10) was used to identify the intersection genes of the two sets and LRGs, and then the "clusterProfiler" package (version 4.14.6) was used to conduct in-depth biological analysis of these core genes. Firstly, the Gene Ontology(GO) analysis of intersecting genes is carried out, which covers three main aspects: Biological Process(BP), Molecular Function(MF) and Cellular Component(CC) to reveal the multiple functions in biology. Then, through the analysis of Kyoto Encyclopedia of Genes and Genomes (KEGG), we further explored the biological pathways involved in these genes, aiming at identifying the metabolic pathways and signal transduction pathways related to DCM.

1.2 GSEA

We analyzed the co-expression of hub genes using Spearman analysis. Gene set enrichment analysis was performed on the Hallmark gene sets (http://software.broadinstitute.org/gsea/msigdb/) using the R package “clusterProfiler” based on the significant co-expression gene of hub genes (P<0.05).

1.3 Masson Staining

Masson’s trichrome staining was performed using a commercial kit (G1006, Servicebio, China) according to the manufacturer’s instructions with minor modifications. Briefly, formalin‑fixed, paraffin‑embedded tissue sections (4 μm thick) were deparaffinized in xylene and rehydrated through a graded ethanol series to distilled water. The sections were then immersed in Masson A solution (mordant) and incubated at 65 °C for 30 min, followed by rinsing under running tap water for 5 min. Meanwhile, Masson D and Masson F solutions were preheated in a 55 °C water bath. For nuclear staining, the sections were placed in a mixed staining solution containing equal volumes of Masson B and Masson C for 1 min at room temperature, then rinsed briefly with tap water. Differentiation was carried out using the supplied differentiation solution for a few seconds (until the background became clear), followed by a tap-water wash. Subsequently, the sections were stained with Masson D solution for 6 min, rinsed with tap water, and then treated with Masson E solution for 1 min. Without washing, the sections were drained briefly and immediately immersed in Masson F solution for 2-30 s (optimal staining time was determined empirically). After a rapid rinse in 1% acetic acid for differentiation, the sections were dehydrated through two changes of absolute ethanol (2 min each), cleared in xylene for 5 min, and mounted with neutral balsam. Images were captured under a light microscope.

1.4 WGA (Wheat germ agglutinin) staining
 Paraffin‑embedded tissue sections (4 μm) were deparaffinized in xylene and rehydrated through a graded ethanol series to distilled water. For antigen retrieval, the sections were immersed in sodium citrate buffer (pH 6.0) and heated in a microwave oven for 10 min, with careful attention to prevent buffer evaporation and drying of the sections. After cooling to room temperature, the slides were washed three times (5 min each) with phosphate‑buffered saline (PBS, pH 7.4) on a rocking platform. A hydrophobic barrier pen was used to encircle each tissue section, and a working solution of WGA (diluted 1:200 in PBS) was applied to cover the tissue. The slides were then incubated in a humidified, light‑protected chamber at 37 °C for 1 h. Following incubation, the slides were washed again with PBS on a rocking platform (three times, 5 min each). Nuclei were counterstained with DAPI solution (1 μg/mL) at room temperature in the dark for 10 min. Finally, the sections were mounted with an anti‑fade mounting medium, and images were acquired using a fluorescence microscope equipped with appropriate excitation/emission filters.

1.5 Reverse transcription-quantitative polymerase chain reaction (RT-PCR)

Total RNA was extracted from cells using Trizol reagent (TAKARA, USA), and RNA concentration was measured using a spectrophotometer. RNA samples (1μg) were reverse transcribed using the PrimeScript™ RT reagent kit (TAKARA, RR047A, JPN). RT-qPCR was performed using the TB Green™ Fast qPCR Mix reagent kit (TAKARA, RR430A, JPN). The Prism Primer sequences were listed in Table S3. Data are expressed as the mean of at least six independent experiments (biological replicates).

1.6 Western blot

Total proteins were extracted from left ventricle using RIPA lysis buffer (AR102‑100, BOSTER) supplemented with 1% protease inhibitor cocktail (AR1178, BOSTER) and 1% phosphatase inhibitor (AR1195, BOSTER). The homogenates were sonicated on ice using an ultrasonic processor with an output power of 30-40% in pulsed mode (10-15s per pulse, three pulses with 15s intervals). After centrifugation at 12,000 g for 10 min at 4 °C, the supernatant was collected, and protein concentrations were determined using a BCA assay. Equal amounts of protein were separated by SDS‑polyacrylamide gel electrophoresis and then electrotransferred onto PVDF or nitrocellulose membranes. The membranes were blocked with 0.5% milk in Tris‑buffered saline containing 0.1% Tween‑20 (TBST) for 1 h at room temperature. Subsequently, the membranes were incubated with primary antibodies at 4 °C for 12-16 h, followed by incubation with horseradish peroxidase (HRP)‑conjugated secondary antibodies at 4 °C for 2 h. Between each step, the membranes were washed thoroughly with TBST (three times, 5 min each). Immunoreactive bands were visualized using enhanced chemiluminescence (ECL) substrates and captured with a Bio‑Rad chemiluminescence imaging system.

Supplementary Tables

Table S1 Instrumental variables derived from eQTLs for 18 candidate genes and their inclusion in two‑sample Mendelian randomization analyses for dilated cardiomyopathy and heart failure.

| Gene | eQTL ID | DCM outcome considered | SNPs retained for DCM MR | Included in DCM MR | HF outcome considered | SNPs retained for HF | Included in HF MR |
| --- | --- | --- | --- | --- | --- | --- | --- |
| *ARID1A* | eqtl-a-ENSG00000117713 | ebi-a-GCST90018614 | 0 | No | ukb-d-I50 | 3 | Yes |
| *IER3* | eqtl-a-ENSG00000137331 | ebi-a-GCST90018614 | 8 | Yes | ukb-d-I50 | 10 | Yes |
| *DEPDC1* | None | 0 | 0 | No | 0 | 0 | No |
| *PRPF6* | eqtl-a-ENSG00000101161 | ebi-a-GCST90018614 | 0 | No | ukb-d-I50 | 3 | Yes |
| *PAXIP1* | eqtl-a-ENSG00000157212 | 0 | 0 | No | 0 | 0 | No |
| *DDX41* | None | 0 | 0 | No | 0 | 0 | No |
| *ANKZF1* | eqtl-a-ENSG00000163516 | 0 | 0 | No | 0 | 0 | No |
| *EXT1* | eqtl-a-ENSG00000182197 | ebi-a-GCST90018614 | 0 | No | ukb-d-I50 | 5 | Yes |
| *GIGYF2* | eqtl-a-ENSG00000204120 | 0 | 0 | No | 0 | 0 | No |
| *EGFR* | None | 0 | 0 | No | 0 | 0 | No |
| *SAFB* | None | 0 | 0 | No | 0 | 0 | No |
| *RARG* | eqtl-a-ENSG00000172819 | 0 | 0 | No | 0 | 0 | No |
| *ALDOB* | eqtl-a-ENSG00000136872 | 0 | 0 | No | 0 | 0 | No |
| *AGRN* | eqtl-a-ENSG00000188157 | 0 | 0 | No | 0 | 0 | No |
| *LAP3* | eqtl-a-ENSG00000002549 | ebi-a-GCST90018614 | 4 | Yes | ukb-d-I50 | 8 | Yes |
| *IRF2BP2* | eqtl-a-ENSG00000168264 | ebi-a-GCST90018614 | 0 | No | ukb-d-I50 | 4 | Yes |
| *ZC3H18* | eqtl-a-ENSG00000158545 | 0 | 0 | No | 0 | 0 | No |
| *TPBG* | eqtl-a-ENSG00000146242 | ebi-a-GCST90018614 | 0 | No | ukb-d-I50 | 3 | Yes |

Table S2 MR-Egger, MR-PRESSO and Cochran's Q test

| Exposure | Outcome | Heterogeneity | | Pleiotropy_test | | MR-PRESSO | |
| --- | --- | --- | --- | --- | --- | --- | --- |
|  |  | Cochran's Q | FDR-adjusted P value | Egger intercept | FDR-adjusted P value | RSSobs | pval |
| EXT1: eqtl-a-ENSG00000182197 | ukb-d-I50 | 4.954138 | 0.292035 | -7.082047e-05 | 0.6999 | 5.175525 | 0.684 |
| IER3: eqtl-a-ENSG00000137331 | ebi-a-GCST90018614 | 6.764063 | 0.343218 | -0.1077539 | 0.1710956 | 9.487744 | 0.2195074 |

Table S3 Primer sequences

| Number | Name | NCBI ID | Sequence |
| --- | --- | --- | --- |
| 1 | *EXT1* | 14042 | F: 5′-AGACATCATGGAGGACCTGGACAC-3′;  R: 5′-TGGGTTCTCATGCAGCTTCTC-3′ |
| 2 | *IER3* | 15937 | F: 5′-AGCTCTGAAGGCAACAGCAACA-3′;  R: 5′-GCTCCATGCAGGTAGCAGGAA-3′ |

Supplementary Figures

Figure S1 The flowchart of Mendelian randomization analysis

Figure S2 The Standardization of datasets GSE3586 and GSE17800

(A) Standardization of datasets GSE3586. (B) Standardization of datasets GSE17800.

Figure S3 Weighted gene co‑expression network analysis (WGCNA) and GO functional enrichment of the lactylation-related genes.

(A-B) The cluster dendrogram showed different module eigengenes. (C) Sample clustering and phenotypic information of the dataset. (D) Biological Processes enrichment analysis. (E) Cellular Component enrichment analysis. (F) Molecular Function enrichment analysis.

Figure S4 Nomogram for predicting DCM risk and its calibration curve.

(A) Nomogram of the DCM risk prediction model. (B) Calibration plot of the nomogram.

Figure S5 Consensus clustering analysis for determining the optimal number of clusters.

(A-E) Consensus clustering matrix when the number of clusters k=2-6. (F) Consensus cumulative distribution function (CDF) graph. (G) Delta area plot (the relative change of area under the CDF curve for k compared with K-1). (H) consensus tracking map.

Figure S6 Validation of *EXT1, IER3* expression and their diagnostic performance in GSE57338 and the expression levels of *ANP*, *BNP*, *TGF-β1*, *Collagen I*, and *Collagen III*.

(A) The expression levels of *EXT1* and *IER3* in the validation set GSE57338. (B) ROC curves of *EXT1* and *IER3* in GSE57338. (C) decision curve analysis diagram. (D) Calibration curve. (E) Statistical chart of the expression levels of *ANP*, *BNP*, *TGF-β1*, *Collagen I*, and *Collagen III*.
